# Methylene Blue Attenuates Lung Injury Induced by Hindlimb Ischemia Reperfusion in Rats

**DOI:** 10.1155/2018/2508620

**Published:** 2018-03-11

**Authors:** Liangrong Wang, Baihui Chen, Bi Lin, Yuzhu Ye, Caiying Bao, Xiyue Zhao, Lida Jin, Xiangqing Xiong

**Affiliations:** Department of Anesthesiology, The First Affiliated Hospital of Wenzhou Medical University, Wenzhou, Zhejiang Province 325000, China

## Abstract

**Objective:**

This study was aimed to investigate the protective effect of methylene blue against lung injury induced by reperfusion of ischemic hindlimb in a rat model.

**Methods:**

Twenty-four healthy adult male Sprague-Dawley rats were equally randomized into three groups: sham (SM) group, ischemia reperfusion (IR) group, and methylene blue (MB) group. Rats in both IR and MB groups were subjected to 4 h of ischemia by clamping the left femoral artery and then followed by 4 h of reperfusion. Treatment with 1% methylene blue (50 mg/kg) was administrated intraperitoneally at 10 min prior to reperfusion in the MB group. After 4 h of reperfusion, malondialdehyde (MDA) level, myeloperoxidase (MPO), and superoxide dismutase (SOD) activities in lung tissue were detected; inflammatory cytokines, including IL-1*β* and IL-6, were measured in bronchoalveolar lavage fluid (BALF); correspondingly, the morphological changes and water content in both gastrocnemius muscle and lung samples were evaluated.

**Results:**

Hindlimb IR caused remarkable morphological abnormalities and edema in both muscle and lung tissues. SOD activity was decreased, both the MPO activity and MDA level in lung tissue, as well as IL-1*β* and IL-6 levels in BALF, were increased in the IR group (*p* < 0.05). Compared with the IR group, SOD activity was increased, whereas MPO activity and MDA level in lung tissue and IL-1*β* and IL-6 levels in BALF were decreased in the MB group (*p* < 0.05). Also, the histological damage and edema in both lung and muscle tissues were significantly attenuated by the treatment of methylene blue.

**Conclusion:**

Methylene blue attenuates lung injury induced by hindlimb IR in rats, at least in part, by inhibiting oxidative stress.

## 1. Introduction

Prolonged cessation of the blood flow in lower extremity would cause ischemic damage, while subsequent restoration of blood flow paradoxically provokes IR injury. It has been well documented that lower limb IR could lead to remote lung injury, which is primarily characterized by increased pulmonary capillary permeability, edema, and neutrophil infiltration [1, 2]. Although the exact mechanisms underlying these processes are not fully understood, oxygen radical formation and oxidative stress are believed to play key roles [3, 4].

Methylene blue is a standard photoacoustic dye, and it is a low-cost drug with lower toxicity that has been useful in cardiopulmonary bypass surgery and in clinical treatment of septic shock, methaemoglobinaemia, and Alzheimer's [5–8]. It is reported that methylene blue transfers electrons from flavoenzymes, primarily xanthine oxidase (XO), where molecular oxygen is normally converted into superoxide radicals; thus, methylene blue could be used as an antioxidative agent [9]. The aim of this study was to determine whether methylene blue would attenuate lung injury induced by hindlimb IR in a rat model.

Here, we modeled hindlimb IR by clamping left femoral artery for 4 h followed by 4 h of reperfusion in rats, and the effects of methylene blue treatment were evaluated. The effects of methylene blue on local muscle and remote lung injury IR were determined histologically. Neutrophil infiltration was predicted by MPO activity and polymorphonuclear cell (PMN) count, whereas the presence of TNF-*α* level in serum and gastrocnemius muscle as well as IL-1*β* and IL-6 levels in BALF was an indicative of inflammation. The measurement of SOD activity and MDA level in lung tissue showed the balance between antioxidant action and oxidation. Tissue water content was also calculated using wet/dry weight (W/D) ratio.

## 2. Materials and Methods

All experimental procedures were performed in accordance to the guidelines of the National Institutes of Health for Care and Use of Laboratory Animal and were approved by the Animal Care and Use Committee of Wenzhou Medical University. Healthy adult male Sprague-Dawley rats weighing 250–300 g were used in this study. The animals were housed in a licensed, climate-controlled research laboratory, with 12-hour light/dark cycle and free access to standard diet and water. All rats were fasted but without water deprivation for 12 h prior to experimental protocol.

### 2.1. Group Allocation

Twenty-four rats were randomized into three groups: SM group, IR group, and MB group, with eight per group. Both the IR and MB groups were subjected to 4 h of ischemia by clamping the left femoral artery followed by 4 h of reperfusion. Treatments with 1% methylene blue (50 mg/kg, approximately 2 ml) and 2 ml of normal saline were administrated intraperitoneally at 10 min prior to reperfusion in the MB and IR groups, respectively. Rats in the SM group were subjected to similar surgical procedures except for arterial occlusion, the rubber band was in place without being inflated, and 2 ml of normal saline was given at the same manner as the IR group.

### 2.2. IR Protocol

Anesthetic mixture consisting of 80 mg/kg ketamine and 5 mg/kg diazepam was administered intraperitoneally for anesthetizing the rats. The rats were then placed in a supine position with a heating pad was applied to keep rectal temperature at 37-38°C throughout the protocol, and fluid loss was replaced with hourly intraperitoneal injection of 0.5 ml normal saline. The left inguinal area was sheared and sterilized, the inner side of the left groin was incised, and the left femoral artery was then isolated and clamped using a single nontraumatic clamp. After the left hindlimb was exsanguinated, a rubber band was applied proximal to the left greater trochanter for 4 h to mimic clinical situation in both the IR and MB groups. Throughout the period of ischemia, the animals were anesthetized with supplemental dose of anesthetic mixture. After 4 h of hindlimb ischemia, both the arterial clamp and rubber band were removed and another 4 h of reperfusion was allowed. At the end of reperfusion, the animals were euthanized by anesthetic overdose, the gastrocnemius muscle and lung tissues were then harvested, and BALF and abdominal aorta blood samples were collected for subsequent analysis.

### 2.3. Histological Examination

The gastrocnemius muscle and lung samples were harvested and immediately fixed in 10% buffered formaldehyde for 24 h and processed according to standard procedures by embedding in paraffin wax. The muscle and lung tissues were sectioned into 5 *μ*m thick slices, deparaffinized, and stained with hematoxylin and eosin for light microscopic analysis. Muscle fiber degeneration, disorganization, and inflammatory cell infiltration were scored to evaluate the extent of muscle injury [10], and the lung injury score was rated using previously described methods [11]. Neutrophil sequestration was quantified by the number of PMNs in the alveolar septal wall and expressed as the mean number of PMNs per 10 high-power fields in an equivalent number of alveoli.

### 2.4. Measurement of Wet/Dry Weight (W/D) Ratio

Tissue edema was evaluated using the W/D ratio calculation. After sampling, the gastrocnemius and lower right lung specimens were immediately weighed to gain wet weight, then samples were dried at a temperature of 80°C for 48 hours, and the dry weight were measured.

### 2.5. Detection of XO Activities and TNF-*α* Level in Skeletal Muscle and Serum

Here, the XO activity was quantified indirectly using uric acid concentration. The gastrocnemius muscle samples for determination of XO activity were prepared as described previously [12]. The levels of uric acid in muscle homogenate and serum were determined using the spectrophotometric assay at 292 nm. An enzyme-linked immunosorbent assay kit (Westang Biotechnology Co. Ltd., Shanghai, China) was used to detect TNF-*α* level according to the manufacturer's instructions.

### 2.6. Measurements of IL-1*β* and IL-6 Levels in BALF

BALF was collected by the method formerly described [13]. Briefly, the lungs were lavaged thrice using 5 ml phosphate-buffered saline through a tracheal cannula and the BALF was harvested. The BALF was then centrifuged at 10,000*g* for 10 min at 4°C. Subsequently, the supernatant was collected and stored at −80°C for further detection. The levels of IL-1*β* and IL-6 in BALF were determined using commercially available enzyme-linked immunosorbent assay kits (Westang Biotechnology Co. Ltd., Shanghai, China) according to the manufacturer's instructions.

### 2.7. Measurements of MPO and SOD Activities and MDA Level in Lung Tissue

After being cut into small segments, each portion of the upper right lung tissues was homogenized in 1 ml 0.1 M phosphate-buffered solution (pH 7.4) using a rotor-stator homogenizer. The homogenate was collected and then centrifuged at 12,000*g* for 30 min at 4°C, and the supernatant fraction was harvested to detect MPO and SOD activities and MDA level. Technically, MPO and SOD activities were measured by O-dianisidine oxidase and xanthine method, respectively, and thiobarbituric acid reaction was used to assess MDA level. The kits (Nanjing Jiancheng Bioengineering Institute, Nanjing, China) were utilized according to the manufacturer's standard protocols.

### 2.8. Statistics Analysis

Statistical analyses were performed with SPSS version 13.0 software (SPSS Inc., Chicago, IL). All data are expressed as mean ± standard deviation, and one-way analysis of variance (ANOVA) and post hoc Bonferroni correction were used to compare differences among groups. A *p* value < 0.05 was considered statistically significant.

## 3. Results

### 3.1. Histological Changes in Muscle and Lung Tissues

**Figure 1:**
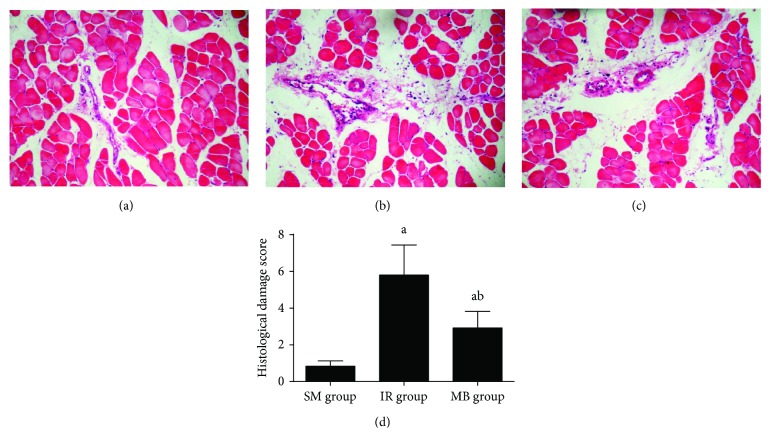
Histological changes in muscle tissue. (a) SM group; (b) IR group; (c) MB group; (d) histological damage score in muscle tissue. ^a^*p* < 0.05 versus SM group; ^b^*p* < 0.05 versus IR group.

No significant damage in skeletal muscle tissue was observed in the SM group (Figure 1(a)). Light microscope examination revealed muscular fiber degeneration, sarcoplasm dissolution, neutrophil infiltration, and erythrocyte diapedesis in muscle tissue from the IR group (Figure 1(b)), and however, skeletal muscle damage was attenuated by methylene blue treatment (Figure 1(c)). As demonstrated, the histological damage score was significantly higher in the IR group than that in the SM group but decreased in the methylene blue treatment group (*p* < 0.05, Figure 1(d)).

**Figure 2:**
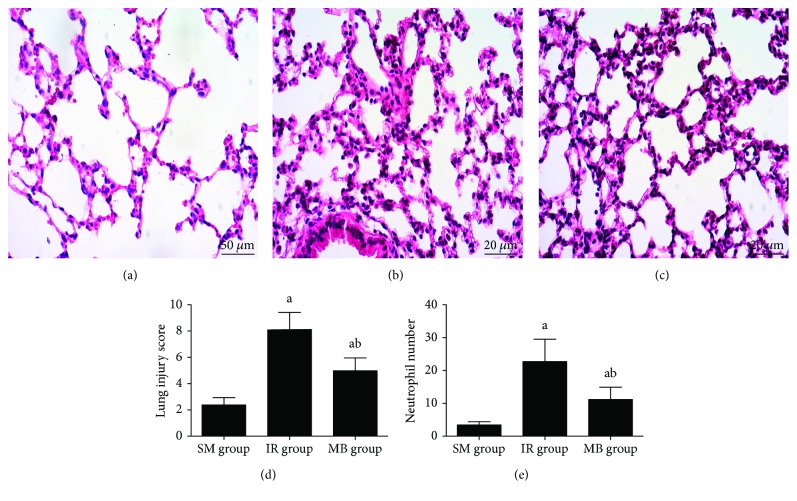
Histological changes in lung tissue. (a) SM group; (b) IR group; (c) MB group; (d) lung injury score; (e) neutrophil number. ^a^*p* < 0.05 versus SM group; ^b^*p* < 0.05 versus IR group.

Histological analysis showed no obvious lung injury or neutrophil infiltration in the SM group (Figures 2(a) and 2(e)), but in contrast, the IR group showed capillary congestion, alveolar hemorrhage, interstitial edema, and neutrophil infiltration in remote lung tissue (Figure 2(b)) and with higher lung injury score and larger neutrophil infiltration number (*p* < 0.05, Figures 2(d) and 2(e)). In comparison with the IR group, the extent of lung injury was alleviated, accompanied with a decrease of lung injury score and reduced neutrophil infiltration number in the MB group (*p* < 0.05, Figures 2(d) and 2(e)).

### 3.2. W/D Ratios in Muscle and Lung Tissues

**Figure 3:**
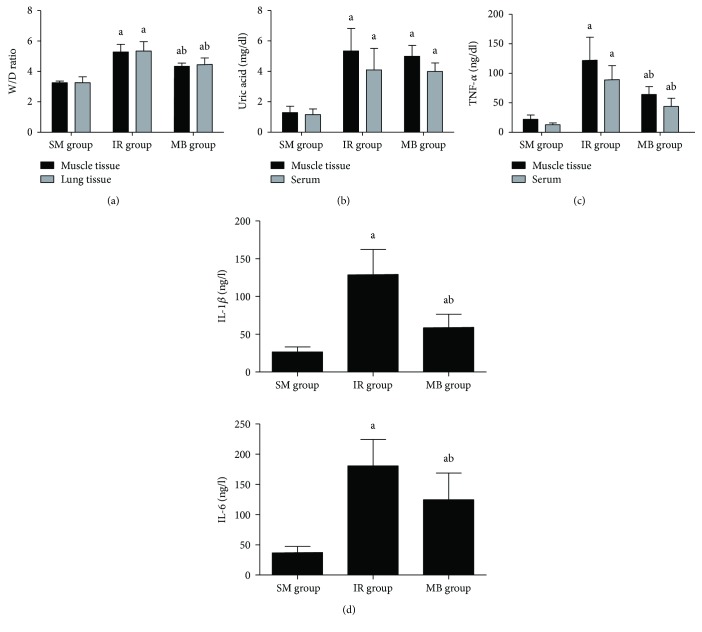
Water content, XO activity, and inflammatory cytokines. (a) Water contents in muscle and lung tissues; (b) uric acid levels in muscle and serum; (c) TNF-*α* level in serum and muscle; (d) IL-1*β* and IL-6 levels in BALF. ^a^*p* < 0.05 versus SM group; ^b^*p* < 0.05 versus IR group.

W/D ratios in both gastrocnemius muscle and lung tissues were increased in the IR group as compared to the SM group (*p* < 0.05), but ratios were lower in the MB group than those in the IR group (*p* < 0.05, Figure 3(a)).

### 3.3. XO Activities in Skeletal Muscle and Serum

Uric acid level was employed to indicate XO activity. As compared to the SM group, a significant increase in uric acid levels in both gastrocnemius muscle and blood was found in the IR and MB groups (*p* < 0.05, Figure 3(b)). No statistical differences of both muscle and serum uric acid levels between the IR and MB groups were found (*p* > 0.05, Figure 3(b)).

### 3.4. TNF-*α* Level in Serum and Muscle

Figure 3(c) shows that TNF-*α* level in both serum and gastrocnemius muscle was significantly higher in both the IR and MB groups than that in the SM group (*p* < 0.05), while methylene blue decreased TNF-*α* level as compared to the IR group (*p* < 0.05).

### 3.5. IL-1*β* and IL-6 Levels in BALF

As demonstrated in Figure 3(d), IL-1*β* and IL-6 levels in BALF were significantly elevated in the IR and MB groups as compared to the SM group (*p* < 0.05), while IL-1*β* and IL-6 levels in BALF were lower in the MB group than those in the IR group (*p* < 0.05).

### 3.6. MPO and SOD Activities and MDA Level in Lung Tissue

**Figure 4:**
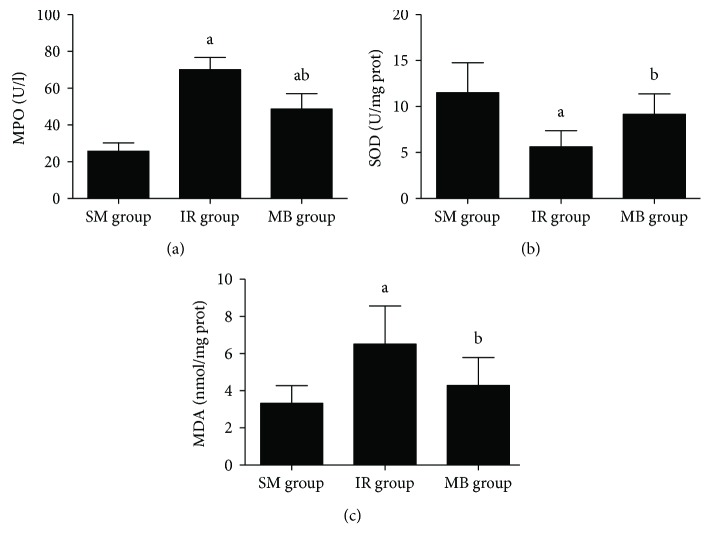
MPO and SOD activities and MDA level in lung tissue. (a) MPO activity in lung tissue; (b) SOD activity in lung tissue; (c) MDA level in lung tissue. ^a^*p* < 0.05 versus SM group; ^b^*p* < 0.05 versus IR group.

MPO activity and MDA level were increased, and SOD activity in lung tissue was decreased in the IR group as compared to the SM group (*p* < 0.05). As shown in Figure 4, methylene blue treatment decreased MPO activity and MDA level and increased SOD activity (*p* < 0.05).

## 4. Discussion

Here, the results demonstrated that treatment with methylene blue not only attenuated histological damage in local muscle tissue but also inhibited oxidative stress, decreased water content, and finally alleviated lung injury remote to hindlimb IR in rats.

The involvement of oxygen radicals in tissue or organ IR injury has been well documented, with XO being proposed as the primary sources of these toxic materials [14, 15]. XO has been regarded as a free radical generator by various cell types including PMNs, endothelial, and epithelial cells. Consequently, it is involved in cellular oxidative stress induced by IR or inflammation [16]. Xanthine dehydrogenase and XO are two separate but interconvertible enzymes involved in oxygen radical production. Ischemia-induced tissue hypoxia has been proven to result in the conversion of xanthine dehydrogenase to XO and breakdown of ATP to hypoxanthine. Hypoxanthine in turn acts as a substrate for XO to produce a mass of superoxide, hydrogen peroxide, hydroxyl radicals, and uric acid during restoration of oxygen supply [16]. Furthermore, reperfusion leads to the systemic release of overproduced oxygen radicals in skeletal muscle causing the attack of polyunsaturated fatty acid in the endothelium cytomembrane, a process also known as lipid peroxidation. This increases the pulmonary vascular endothelium permeability to fluids and inflammatory cells, ultimately resulting in pulmonary edema and neutrophil sequestration in lung tissue [14, 17, 18]. Also, free radicals directly induce PMN activation and adhesion to vascular endothelium [14, 15, 19], which in turn causes a vicious cycle releasing other free radicals, complements, proteases, and proinflammatory cytokines, leading to further lung injury [19].

MDA, the end product of peroxidized polyunsaturated fatty acids, is an indicative of lipid peroxidation, while SOD catalyzes the transformation of superoxides to hydrogen peroxide and subsequently into water and oxygen. Increased MPO activity is an indicator of neutrophil activation because MPO is found almost exclusively within neutrophils [20]. The proinflammatory cytokines including TNF-*α*, IL-1*β*, and IL-6 are prominent in lung injury especially following hindlimb IR [1, 21]. As demonstrated in our results, hindlimb IR in rat caused gastrocnemius muscle damage and morphologic abnormalities in remote lung tissue, accompanied by pulmonary edema and neutrophil infiltration. Additionally, hindlimb IR increased MDA level and decreased SOD activity in lung tissue, whereas proinflammatory cytokines including TNF-*α* level in serum and muscle, IL-1*β*, and IL-6 levels in BALF were significantly enhanced after hindlimb IR occurrence. These results confirmed that oxidative stress plays vital roles in lung injury induced by hindlimb IR; therefore, alleviating or attenuating the oxygen radical production might be an alternative therapy with potential efficacy.

Due to its low toxicity and powerful penetration capacity, the thiazine cationic dye methylene blue is commonly used for lymph nodal vital staining. Furthermore, methylene blue is now represented as a new class of antioxidant drugs, and it is currently used in treating methemoglobinemia, cyanide and carbon monoxide poisoning, and some neurological diseases, with less side effects and low cost [6, 7, 22]. The effects of methylene blue in attenuating IR injury in several tissues or organs have been documented [23–27]. As indicated in various studies, oxygen radicals are produced by the iron-sulfuric center of XO that is known to serve as an oxidative site; however, by acting as an alternative receptor of electrons, methylene blue competes with molecular oxygen for the transference of the pair electrons from the iron-sulfuric site of XO, thereby inhibiting the production of superoxide radicals and hydroxyl radicals while allowing the metabolism of xanthine to uric acid [28]. Methylene blue has been reported to increase the activity of antioxidant enzymes such as SOD and suppress oxygen radical formation in a dose-dependent manner, and the efficacy was similar to SOD in models of liver and kidney IR [9, 27, 29–32]. It is also known to inhibit the activation of neutrophils, decrease the expression of adhesive molecules, and limit the overproduction of inflammatory cytokines [9, 28, 33]. Methylene blue has also been proven to exert protective effects against IR injury by other mechanisms not restricted to its inhibition on the production of superoxide by competing directly with molecular oxygen [34]. Permeating through membranes, methylene blue accumulates within the mitochondria and improves mitochondrial respiration by minimizing electron leakage from the electron transport chain and reducing superoxide formation during reperfusion [7, 35]. Previous studies have also documented that methylene blue could stabilize energy metabolism and increase ATP synthesis after reoxygenation, whereas activating hypoxia inducible factor-1/erythropoietin signaling pathway [7, 35, 36]. Additionally, methylene blue may decrease nitric oxide (NO) formation by inhibiting inducible nitric oxide synthase, which in turn decrease the production of toxic radical peroxynitrite and peroxynitrous acid [17, 37]. This may also explain the protective effects of methylene blue in suppressing lipid peroxidation and inflammation activation during reperfusion. However, in the context of hindlimb IR injury, the exact mechanisms involving in the protective effects of methylene blue still need detailed studies.

As expected, our results showed that methylene blue treatment attenuated both local skeletal muscle and remote lung injury in rats with hindlimb IR, which was evidenced by improved morphological manifestations, alleviated tissue edema, and reduced neutrophil infiltration; methylene blue also reduced circulatory TNF-*α* level as well as IL-1*β* and IL-6 levels in BALF, decreased MPO activity and MDA level, and increased SOD activity in lung tissue. Thus, the results suggested that methylene blue might exert its protective effects against lung injury induced by hindlimb IR injury by suppressing oxidative stress. As hypoxanthine would be transformed into uric acid with the action of XO, therefore, detection of uric acid level has been proposed to indirectly indicate XO activity [27, 38]. In contrast to classical purine analogue-type XO inhibitors, which could inhibit hypoxanthine and xanthine conversion to uric acid, methylene blue would allow the oxidation of hypoxanthine or xanthine but selectively prevent superoxide formation by acting as competitive alternative cosubstrates. Thus, methylene blue treatment prior to reperfusion showed no significant changes in uric acid levels in gastrocnemius muscle tissue or serum in our study, which was consistent with the results of the previous study [28].

Although our studies show promise, rat models have some limitations in mimicking clinical situation. Particularly, ischemia duration applied in rats is extremely longer than the tourniquet duration in clinical setting [39]. Further studies evaluating the dose-response effect of methylene blue are needed to identify the “ideal” dose; furthermore, the effect of different administration timing and treatment duration should be evaluated. Methylene blue has been demonstrated to prevent hemodynamic instability after hepatic IR [40]; consequently, the monitoring of hemodynamic parameters during the experimental protocol might be helpful to understand its action. Finally, further studies on the precise mechanisms by which methylene blue may attenuate lung injury induced by hindlimb IR are essential to optimize clinical use of methylene blue to maximize its beneficial effects.

## 5. Conclusion

In conclusion, at least in part by inhibiting oxidative stress, treatment with methylene blue attenuates lung injury induced by hindlimb IR in rats.
